# Transpedicular lower vertebral space osteotomy for thoracolumbar kyphosis secondary to old osteoporotic vertebral compression fractures

**DOI:** 10.3389/fsurg.2026.1620511

**Published:** 2026-03-12

**Authors:** YuDong Xia, Li Li, Jie Qin

**Affiliations:** Department of Spine and Traumatology, People's Hospital of Changshou, Chongqing, China

**Keywords:** osteoporosis, osteoporotic vertebral compression fracture, pedicle subtraction osteotomy, thoracolumbar kyphosis, transpedicular lower vertebral space osteotomy

## Abstract

**Background:**

Despite rapid developments in spinal osteotomy techniques, many inevitable short- and long-term complications still occur. The objective of this study is to evaluate the clinical efficacy of transpedicular lower vertebral space osteotomy for the treatment of thoracolumbar kyphosis secondary to long-standing osteoporotic vertebral compression fractures and to explore more effective surgical methods to guide clinical practice.

**Methods:**

We retrospectively analyzed the data of patients who were diagnosed with thoracolumbar kyphosis secondary to long-standing osteoporotic vertebral compression fractures and who underwent corrective surgery at our hospital between January 2014 and December 2020. The patients were divided into two groups: the pedicle subtraction osteotomy (PSO) group (*n* = 16) and the transpedicular lower vertebral space osteotomy group (*n* = 11). Operation time, bleeding volume, postoperative complications, and postoperative drainage were documented. Clinical outcomes were evaluated using the visual analog scale (VAS) score, the Oswestry Disability Index (ODI), and the kyphotic Cobb angle.

**Results:**

During follow-up, it was found that there was no significant difference in the average degree of kyphosis correction between the PSO group and the transpedicular lower vertebral space osteotomy group. Compared with the corresponding preoperative values, the ODI and VAS scores in both groups showed significant improvement, while the complication rate was similar between the groups, and bony fusion was observed at the last follow-up.

**Conclusion:**

Transpedicular lower vertebral space osteotomy was associated with shorter operative time and lower blood loss than PSO, while achieving comparable radiographic correction and clinical outcomes at the last available follow-up. Complication rates were not significantly different between groups. Larger studies with longer follow-up are warranted.

## Introduction

The thoracolumbar spine is the most common site of spinal fractures, but approximately two-thirds to three-quarters of vertebral fractures are clinically undetected (1). A lack of or inappropriate treatment may lead to a progressive collapse of the fractured vertebrae, resulting in local kyphosis, especially in patients with osteoporotic vertebral compression fractures. As the kyphosis progresses, patients may present with intractable thoracolumbar pain, lower limb weakness, urinary and bowel dysfunction, dyspnea, abdominal distension and pain, and other nerve or chest and abdominal organ compression symptoms, which may also cause serious mental health problems. Patients typically seek further treatment to relieve pain and improve the quality of life (12).

Vertebral compression fractures are the most common osteoporotic fractures and are frequently under-recognized, with only a minority being clinically diagnosed (3, 4). Inadequately treated or progressive fractures may lead to vertebral height loss and segmental hyperkyphosis, which is associated with persistent pain, impaired activities of daily living, reduced quality of life, and increased healthcare utilization (4, 5). Hyperkyphosis may also reduce thoracic volume and pulmonary function, contributing to broader functional decline (6). Therefore, for patients with long-standing osteoporotic vertebral compression fractures complicated by fixed thoracolumbar kyphosis and disabling symptoms, surgical correction is often required to relieve symptoms, restore sagittal alignment, and improve function (7, 8).

Since Smith‒Petersen et al. (9) first reported the use of Smith‒Petersen osteotomy (SPO) for the treatment of thoracolumbar kyphosis caused by ankylosing spondylitis, remarkable progress has been made in the treatment of kyphosis because of improvements in imaging and surgical instruments and rapid developments in spinal osteotomy techniques. Chang et al. (10) classified spinal osteotomies into open-wedge osteotomy, closing-wedge osteotomy, and closing‒opening wedge osteotomy. Schwab et al. (11) summarized and classified spinal osteotomy techniques on this basis, including classes such as partial facet joint resection, complete facet joint resection, pedicle and partial body resection, pedicle, partial body, and disc resection; complete vertebra and disc resection; and multiple adjacent vertebrae and disc resection. In clinical applications, the correction achieved at each stage is approximately 10° per classification. If a greater osteotomy angle is needed, a combined multistage osteotomy procedure can be performed.

In clinical practice, the choice of osteotomy depends on deformity rigidity, required correction angle, and patient-specific risk factors. SPO and Ponte osteotomy are commonly used for flexible deformities requiring modest correction, whereas PSO provides larger angular correction but is associated with longer operative time and substantial blood loss. For severe, rigid angular kyphosis, vertebral column resection can achieve powerful correction but carries a high complication burden.

Importantly, these procedures are often performed in elderly patients with osteoporosis, in whom reduced bone quality increases the risks of screw loosening, fixation failure, non-union, and revision surgery, while extensive osteotomy surfaces may further increase bleeding and perioperative morbidity. Therefore, optimizing corrective osteotomy strategies to balance correction efficacy and perioperative safety remains a key clinical challenge in osteoporotic populations.

Despite advances in deformity correction, perioperative morbidity and mechanical complications remain substantial, particularly in osteoporotic patients who are vulnerable to fixation failure and delayed fusion. Therefore, there is a clinical need for an osteotomy strategy that can provide sufficient correction, while minimizing operative time, blood loss, and complication risk in this population. To address this gap, we developed a novel technique—transpedicular lower vertebral space osteotomy—and performed a retrospective comparison with PSO conducted by the same surgical team. This study aimed to evaluate the radiographic correction, functional recovery, and perioperative safety of transpedicular lower vertebral space osteotomy in patients with thoracolumbar kyphosis secondary to long-standing osteoporotic vertebral compression fractures.

## Methods

### Design

We retrospectively analyzed the data of patients who were diagnosed with thoracolumbar kyphosis secondary to long-standing osteoporotic vertebral compression fractures and who underwent corrective surgery at our hospital between January 2014 and December 2020. The following inclusion criteria were used: (1) thoracolumbar kyphosis secondary to long-standing osteoporotic vertebral compression fracture, with a sagittal plane Cobb angle greater than 20°; (2) severe thoracolumbar back pain [visual analogue scale (VAS) score >7] or neurological dysfunction; and (3) ineffective conservative treatment for at least 3 months. Conservative management included analgesic therapy, activity modification, and thoracolumbar bracing, combined with standard antiosteoporosis management. Failure of conservative treatment was defined as persistent severe back pain (VAS >7) and/or neurological dysfunction after at least 3 months of conservative management, with no clinical improvement and/or radiographic progression of kyphosis, resulting in the need for surgical correction.

The exclusion criteria were as follows: (1) degenerative or congenital kyphosis; (2) thoracolumbar spine neoplasm or infection; (3) previous spinal surgery; suspected pathological fracture due to tumor, infection, or other non-osteoporotic etiologies, based on clinical evaluation and imaging (and biopsy when indicated); (4) poor basic condition of the whole body and inability to tolerate surgery; (5) a postoperative follow-up period of less than 3 months; (6) severe degenerative spinal disease (e.g., advanced degenerative scoliosis, severe spinal stenosis, or high-grade spondylolisthesis) that could substantially affect outcomes; and (7) neurogenic diseases or other conditions that could confound pain/function assessment. All procedures involving human participants were conducted in accordance with the institution's ethical standards and the 1964 Declaration of Helsinki, as well as its subsequent amendments or comparable ethical standards. Informed consent was waived because of the retrospective nature of the study. The Medical Ethics Committee of the First Affiliated Hospital of Chongqing Medical University approved this study. As this was a retrospective exploratory study, no *a priori* sample size calculation was performed; all consecutive eligible patients during the study period were included.

### Patients

A total of 27 patients with thoracolumbar kyphosis secondary to long-standing osteoporotic vertebral compression fractures were included in this study. Group A (transpedicular lower vertebral space osteotomy group) consisted of 11 patients aged 50–77 years (mean, 61.3 years). Nine patients had a definite history of trauma: four due to a fall injury, one due to strenuous activity, one due to lumbar sprain, and three due to a weight-bearing injury; two patients had no obvious cause for their trauma. Group B (PSO group) consisted of 16 patients aged 33–74 years (mean, 55.9 years). Twelve patients had a definite history of trauma: nine due to a fall injury, one due to a traffic accident injury, one due to a heavy object injury, and one due to a weight-bearing injury; four patients had no apparent cause of their trauma. Routine preoperative evaluations to determine the steps of the operation included whole-spine and injured-vertebra X-ray imaging, CT scans, 3D reconstructions, and MRI examinations. In addition, the patients' underlying conditions were controlled to minimize the risks involved in the operation.

The average age, body mass index (BMI), lumbar bone mineral density (BMD), sex, affected level, average time from initial fracture to admission, and VAS score for back pain are presented in Table 1. Lumbar bone mineral density was assessed using dual-energy X-ray absorptiometry (DXA) at the lumbar spine (L1–L4). The DXA-derived T-score for the lumbar spine (L1–L4) was recorded for each patient and used to characterize osteoporosis severity. Major comorbidities and chronic medication use were reviewed and optimized preoperatively; however, detailed comorbidity/medication variables were not consistently available for between-group statistical comparisons because of the retrospective nature of the study.

**Table 1:** Baseline characteristics.

Variable	Group A (*n* = 11)	Group B (*n* = 16)	*P*-value (test)
No. of patients	11	16	–
Sex, female	9 (81.8%)	9 (56.2%)	0.231 (Fisher)
Sex, male	2 (18.2%)	7 (43.8%)	–
Age (years)	61.3 ± 9.4 (50.0–77.0)	55.9 ± 11.6 (33.0–74.0)	0.212 (*t*-test)
BMI (kg/m^2^)	21.92 ± 3.64 (17.80–30.82)	23.26 ± 2.51 (18.51–28.32)	0.269 (*t*-test)
Course (years)	9.0 (7.5–13.0) [2.0–27.0]	10.0 (5.8–15.5) [3.0–30.0]	0.961 (Mann–Whitney *U*)
Follow-up (months)	13.0 (7.5–17.0) [6.0–56.0]	10.5 (4.0–19.8) [3.0–42.0]	0.458 (Mann–Whitney *U*)
Lumbar spine T-score (DXA, L1–L4)	−3.34 ± 1.89 (−5.70–1.10)	−3.67 ± 1.06 (−5.70 to −1.50)	0.565 (*t*-test)
Affected level: T11	0 (0.0%)	2 (12.5%)	–
Affected level: T12	3 (27.3%)	5 (31.2%)	–
Affected level: L1	7 (63.6%)	8 (50.0%)	–
Affected level: L2	1 (9.1%)	1 (6.2%)	–
Affected region: Thoracic (T11–T12)	3 (27.3%)	7 (43.8%)	–
Affected region: Lumbar (L1–L2)	8 (72.7%)	9 (56.2%)	0.448 (Fisher)

Continuous variables are presented as mean ± SD (range) when they are normally distributed in both groups; otherwise, they are presented as median (IQR) [range]. Categorical variables are presented as *n* (%). The affected region is analyzed as Thoracic (T11–T12) vs. Lumbar (L1–L2) using Fisher's exact test because of sparse counts in some levels.

### Surgical techniques

After tracheal intubation and general anesthesia, the patient was placed in the prone position. Somatosensory and motor evoked potentials were routinely monitored throughout the operation. The posterior median incision was centered on the injured vertebrae, and the lamina and spinous process were exposed.

#### Group A

**Figure 1:**
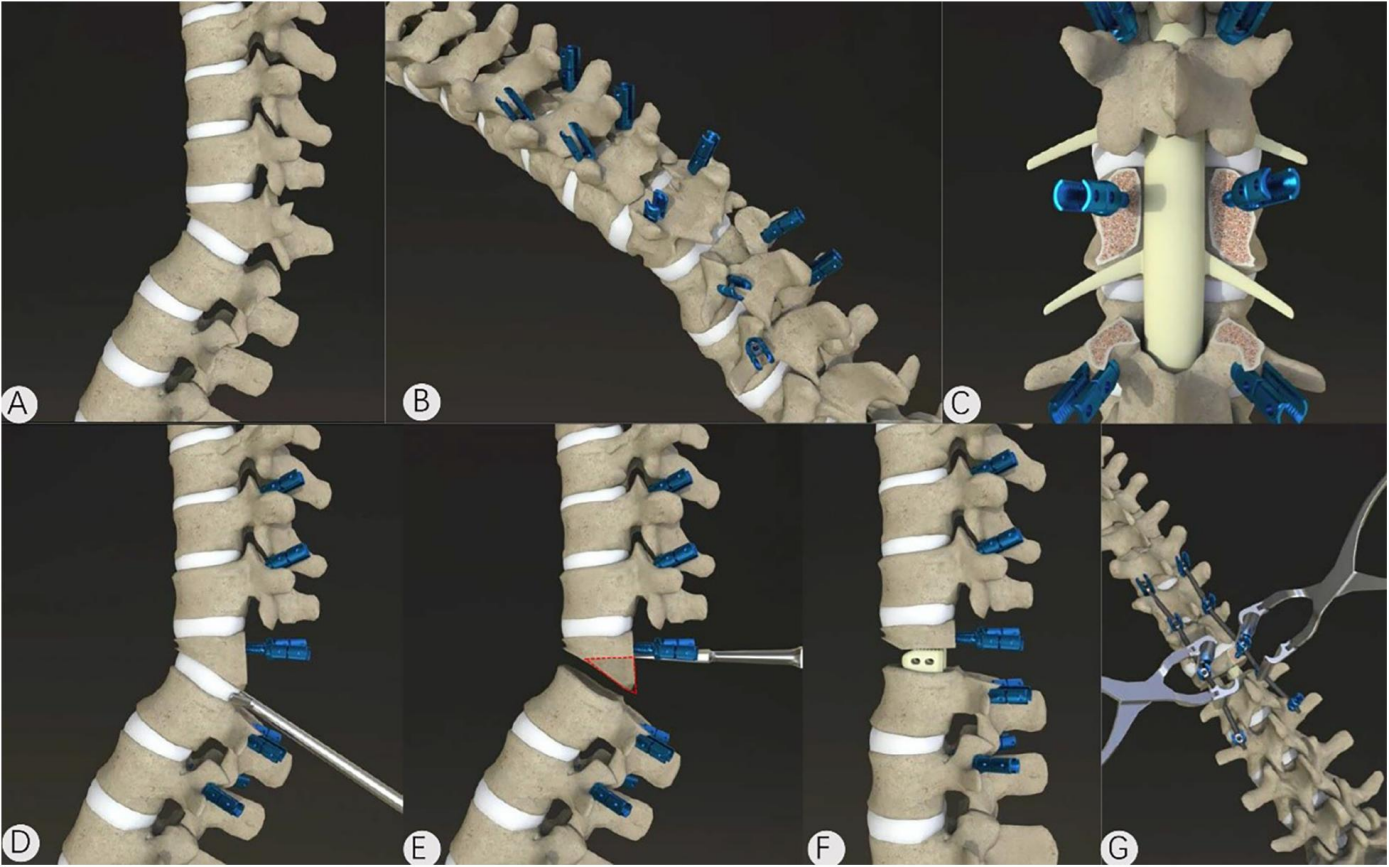
A schematic diagram of post-traumatic thoracolumbar kyphosis treated by transpedicular lower vertebral space osteotomy. **(A,B)** A posterior median incision is used to expose the lamina and spinous process, and screws are placed. **(C)** The lamina is removed, and decompression is performed. **(D)** The part of the vertebral body under the pedicle of the injured vertebra is removed. **(F,G)** Implantation of bone fragments or intervertebral cages and fusion under pressure.

Pedicle screws were implanted into the vertebral body at the osteotomy plane and into 2–3 vertebral bodies above and below the injured vertebrae. Cement augmentation was considered in patients with severe osteoporosis (lumbar spine T-score ≤−2.5) and/or poor intraoperative screw purchase. Polymethylmethacrylate cement was injected through cannulated pedicle screws under fluoroscopic guidance, and injection was stopped immediately if cement leakage was suspected. Because this was a retrospective study, detailed cement volume per screw and injection-related parameters were not consistently documented for all cases and were therefore not included in the comparative analysis. The spinous process, lamina, and articular processes of the injured vertebrae were removed, and the inferior intervertebral disc tissues were exposed and thoroughly removed. Repeated excessive traction of the spinal cord was avoided during the procedure, and temporary fixation rods were inserted to minimize the effect of surgical vibration on the spinal cord. A bone knife was used to remove the part of the vertebral body under the pedicle of the injured vertebra, and a curette was used to repair the osteotomy surface. The same procedure was used on the opposite side to flush the intervertebral space. Interbody fusion cages or autogenous bone, such as the spinous process and lamina removed during the operation, were implanted into the intervertebral space with a titanium rod and fixed under pressure. See Figure 1 for a schematic diagram of the procedure.

#### Group B

Pedicle screws were implanted into 2–4 vertebral bodies above and below the injured vertebrae. Cement augmentation was considered in patients with severe osteoporosis (lumbar spine *T*-score ≤−2.5) and/or poor intraoperative screw purchase. Polymethylmethacrylate cement was injected through cannulated pedicle screws under fluoroscopic guidance, and injection was stopped immediately if cement leakage was suspected. Because this was a retrospective study, detailed cement volume per screw and injection-related parameters were not consistently documented for all cases and were therefore not included in the comparative analysis. The lamina, spinous process, and articular process of the injured vertebrae were removed, and the pedicle and vertebral body were resected through a V-shaped osteotomy to achieve bilateral penetration. Finally, the cortical bone at the posterior edge of the vertebral body was removed, and the V-shaped osteotomy was closed by postural change and local pressure. A titanium rod was implanted and fixed under pressure.

### Postoperative treatment

Routine postoperative pain management, infection prophylaxis, and other symptomatic treatments were used to control primary medical complications. The negative-pressure drainage tube was removed when the postoperative drainage volume was less than 40 mL/day. One week after the operation, the patients were allowed to leave their beds while wearing a thoracolumbar brace. Postoperatively, all patients received antiosteoporosis management according to an institutional protocol, including calcium and vitamin D supplementation and a bisphosphonate when not contraindicated (12). The specific agent, dose, initiation timing, and duration were individualized by the treating physician. Medication use was reviewed during follow-up based on outpatient prescription records and patient self-report; however, detailed regimen information was not consistently available for all patients because of the retrospective nature of this study and was therefore not included in between-group statistical comparisons. Final follow-up was defined as the last available visit with complete clinical and radiographic assessments for each patient, and follow-up duration (months) was recorded.

### Imaging, neurological function, and pain evaluation

Operative time, intraoperative blood loss, postoperative drainage, and complications were recorded. Whole-spine and local X-ray imaging of the operative area was performed preoperatively, postoperatively, and at the final follow-up. The local kyphosis Cobb angle was measured, and the degree of correction was calculated as follows: preoperative Cobb angle−Cobb angle at the final follow-up. All parameters were measured three times by orthopedic residents, and the average value was calculated. The Oswestry Disability Index (ODI) and VAS score were recorded preoperatively, postoperatively, and at the final follow-up to evaluate functional recovery and pain relief. Blinded evaluators assessed both the ODI and the VAS.

### Statistical analysis

Statistical analyses were performed using SPSS (version 26.0; IBM, Chicago, IL, USA). Continuous variables were assessed for normality using the Shapiro–Wilk test and for homogeneity of variances using Levene's test. Normally distributed data were presented as mean ± standard deviation (SD) and compared between groups using the independent-samples *t*-test; when variances were unequal, Welch's *t*-test was used. Non-normally distributed data were presented as median (interquartile range, IQR) and were compared using the Mann–Whitney *U*-test. Categorical variables were presented as *n* (%) and were compared using Fisher's exact test (or the chi-square test when appropriate).

For repeated-measure outcomes, within-group changes from preoperative assessment to final follow-up were analyzed using the paired *t*-test (or the Wilcoxon signed-rank test for non-normal data). Between-group differences in improvement were assessed using change scores (Δ = final follow-up−preoperative) for the ODI and the VAS and Cobb correction (preoperative Cobb angle−final follow-up Cobb angle) for radiographic outcomes. To control type I error across the three main clinical endpoints (Cobb correction, ΔVAS, and ΔODI), *P*-values were adjusted using the Holm–Bonferroni method; other perioperative comparisons were considered exploratory. All tests were two-sided, and a value of *P* < 0.05 was considered statistically significant.

## Results

### Surgical results

All surgeries were completed, and no patient required multisegmental osteotomy. The length of hospital stay was comparable between groups [Group A: 16.0 (13.5–20.0) days vs. Group B: 18.0 (12.0–22.0) days; Mann–Whitney *U*-test, *P* = 0.843]. The number of instrumented fusion segments also did not differ between the groups [Group A: 6.0 (5.0–6.0) vs. Group B: 5.0 (5.0–7.0); Mann–Whitney *U*-test, *P* = 0.609].

Operation time was significantly shorter in Group A than in Group B [189 ± 20 (140–215) min vs. 235 ± 58 (160–335) min; Welch's *t*-test, *P* = 0.008]. Intraoperative blood loss was significantly lower in Group A [200 (200–350) mL] than in Group B [450 (300–800) mL; Mann–Whitney *U-*test, *P* = 0.008]. Postoperative drainage did not differ significantly between the groups [Group A: 570 [290–765] mL vs. Group B: 730 [425–1,028] mL; Mann–Whitney *U*-test, *P* = 0.246] (Table 2).

**Table 2:** Perioperative outcomes.

Variable	Group A (*n* = 11)	Group B (*n* = 16)	*P*-value (test)
Hospital stay (days)	16.0 (13.5–20.0) [12.0–50.0]	18.0 (12.0–22.0) [10.0–28.0]	0.843 (Mann–Whitney *U*)
Instrumented fusion segments	6.0 (5.0–6.0) [4.0–7.0]	5.0 (5.0–7.0) [4.0–8.0]	0.609 (Mann–Whitney *U*)
Operation time (min)	189 ± 20 (140–215)	235 ± 58 (160–335)	0.008 (Welch *t*-test)
Blood loss (mL)	200 (200–350) [100–500]	450 (300–800) [200–1,500]	0.008 (Mann–Whitney *U*)
Drainage (mL)	570 (290–765) [160–1,500]	730 (425–1,028) [320–1,835]	0.246 (Mann–Whitney *U*)
Any complication, *n* (%)	1 (9.1%)	7 (43.8%)	0.090 (Fisher's exact)

Between-group comparisons used the *t*-test or Welch *t*-test for normally distributed variables and the Mann–Whitney *U*-test for non-normally distributed variables. Normality and homogeneity of variance were assessed using Shapiro–Wilk and Levene's tests, respectively.

### Imaging evaluation results

There were no significant between-group differences in the Cobb angle either preoperatively (55.5° ± 14.6° vs. 46.7° ± 10.8°; independent-samples *t*-test, *P* = 0.086) or at the last available follow-up (22.8° ± 10.5° vs. 15.9° ± 8.5°; independent-samples *t*-test, *P* = 0.071). Postoperative Cobb angles were also comparable between groups [median 19.0° (IQR 11.4–23.0) vs. 10.9° (IQR 7.7–13.5); Mann–Whitney *U*-test, *P* = 0.098].

Within each group, the Cobb angle improved significantly from baseline to final follow-up (paired *t*-test, both *P* < 0.001). The mean correction (preoperative minus final follow-up) was 32.7 ± 7.3° in Group A and 30.9 ± 7.2° in Group B, with no significant difference between the groups (independent-samples *t*-test, *P* = 0.533) (Table 3).

**Table 3:** Radiographic outcomes (Cobb angle).

Variable	Group A (*n* = 11)	Group B (*n* = 16)	*P*-value (test)
Cobb angle preop (°)	55.5 ± 14.6 (36.9–78.0)	46.7 ± 10.8 (23.9–66.1)	0.086 (*t*-test)
Cobb angle postop (°)	19.0 (11.4–23.0) [3.5–32.5]	10.9 (7.7–13.5) [1.1–33.5]	0.098 (Mann–Whitney *U*)
Cobb angle final follow-up (°)	22.8 ± 10.5 (4.8–39.3)	15.9 ± 8.5 (2.0–31.9)	0.071 (*t*-test)
Cobb correction (°)	32.7 ± 7.3 (23.4–42.9)	30.9 ± 7.2 (16.6–42.8)	0.533 (*t*-test)
Within-group change (preop. vs. final), *P*	<0.001 (paired *t*-test)	<0.001 (paired *t*-test)	–

Cobb correction was calculated as the preoperative Cobb angle minus the final follow-up Cobb angle. Within-group comparisons (preoperative vs. final follow-up) are shown in the last row.

### Complications

No serious complications such as spinal cord injury, major vessel trauma, or death occurred in either group. In Group A, one patient developed a surgical wound infection after the procedure but recovered well after active secondary surgical debridement. In Group B, four patients experienced hypoalbuminemia or moderate anemia after the operation, one patient had abdominal distension, one had delirium, and one developed a surgical site hematoma that was promptly treated with hematoma removal surgery. All patients with complications recovered well after active treatment. Overall, 1/11 (9.1%) patients in Group A and 7/16 (43.8%) patients in Group B experienced at least one postoperative complication. The overall complication rate did not differ significantly between the groups (Fisher's exact test, *P* = 0.090).

### Follow-up results

All 27 patients were followed up for 3–56 months (mean 15.2 months). Final follow-up was defined as the last available visit with complete clinical and radiographic assessments for each patient.

At the last available follow-up, VAS and ODI scores were comparable between the two groups [VAS: 2 (1–2) vs. 2 (1–3), Mann–Whitney *U*-test, *P* = 0.609; ODI: 12.0 (7.0–16.0)% vs. 13.0 (7.5–19.0)%, Mann–Whitney *U*-test, *P* = 0.862]. Both groups showed significant within-group improvements from preoperative assessment to final follow-up (all *P* < 0.001). Between-group comparisons based on change scores (Δ = final follow-up-preoperative) showed no significant differences in VAS improvement [ΔVAS: −7.0 (−7.5 to −5.0) vs. −5.0 (−6.2 to −5.0), *P* = 0.227]. ODI improvement was greater in Group A in unadjusted analysis (ΔODI: −56.7 ± 16.5 vs. −43.8 ± 14.1, *t*-test, *P* = 0.038), but this difference did not remain significant after a Holm–Bonferroni correction was performed across the three main endpoints (Cobb correction, ΔVAS, and ΔODI) (adjusted *P* = 0.114) (Table 4).

**Table 4:** Functional outcomes (VAS and ODI).

Variable	Group A (*n* = 11)	Group B (*n* = 16)	*P*-value (test)
VAS preoperative	8 (8–9) [6–10]	8 (7–8) [1–9]	0.134 (Mann–Whitney *U*)
VAS postoperative	4 ± 2 (2–8)	3 ± 2 (0–6)	0.247 (*t*-test)
VAS final follow-up	2 (1–2) [1–3]	2 (1–3) [0–4]	0.609 (Mann–Whitney *U*)
VAS change Δ (final-pre)	−7.0 (−7.5 to −5.0) [−9.0 to −4.0]	−5.0 (−6.2 to −5.0) [−8.0–3.0]	0.227 (Mann–Whitney *U*)
VAS within-group P (preop. vs. final)	<0.001 (paired *t*-test)	<0.001 (Wilcoxon signed-rank)	–
ODI preoperative (%)	70.4 ± 15.3 (44.0–92.0)	57.8 ± 18.0 (20.0–86.0)	0.069 (*t*-test)
ODI postoperative (%)	32.0 (30.0–49.0) [24.0–54.0]	30.0 (25.0–37.5) [6.0–46.0]	0.151 (Mann–Whitney *U*)
ODI final follow-up (%)	12.0 (7.0–16.0) [6.0–36.0]	13.0 (7.5–19.0) [0.0–34.0]	0.862 (Mann–Whitney *U*)
ODI change Δ (final-pre)	−56.7 ± 16.5 (−80.0 to −22.0)	−43.8 ± 14.1 (−68.0 to −18.0)	0.038 (*t*-test)
ODI within-group P (preop. vs. final)	<0.001 (paired *t*-test)	<0.001 (paired *t*-test)	–

The change score Δ was calculated as the final follow-up minus preoperative value. ODI values are expressed as percentage points. Within-group comparisons (preoperative vs. final follow-up) are reported for each outcome.

**Figure 2:**
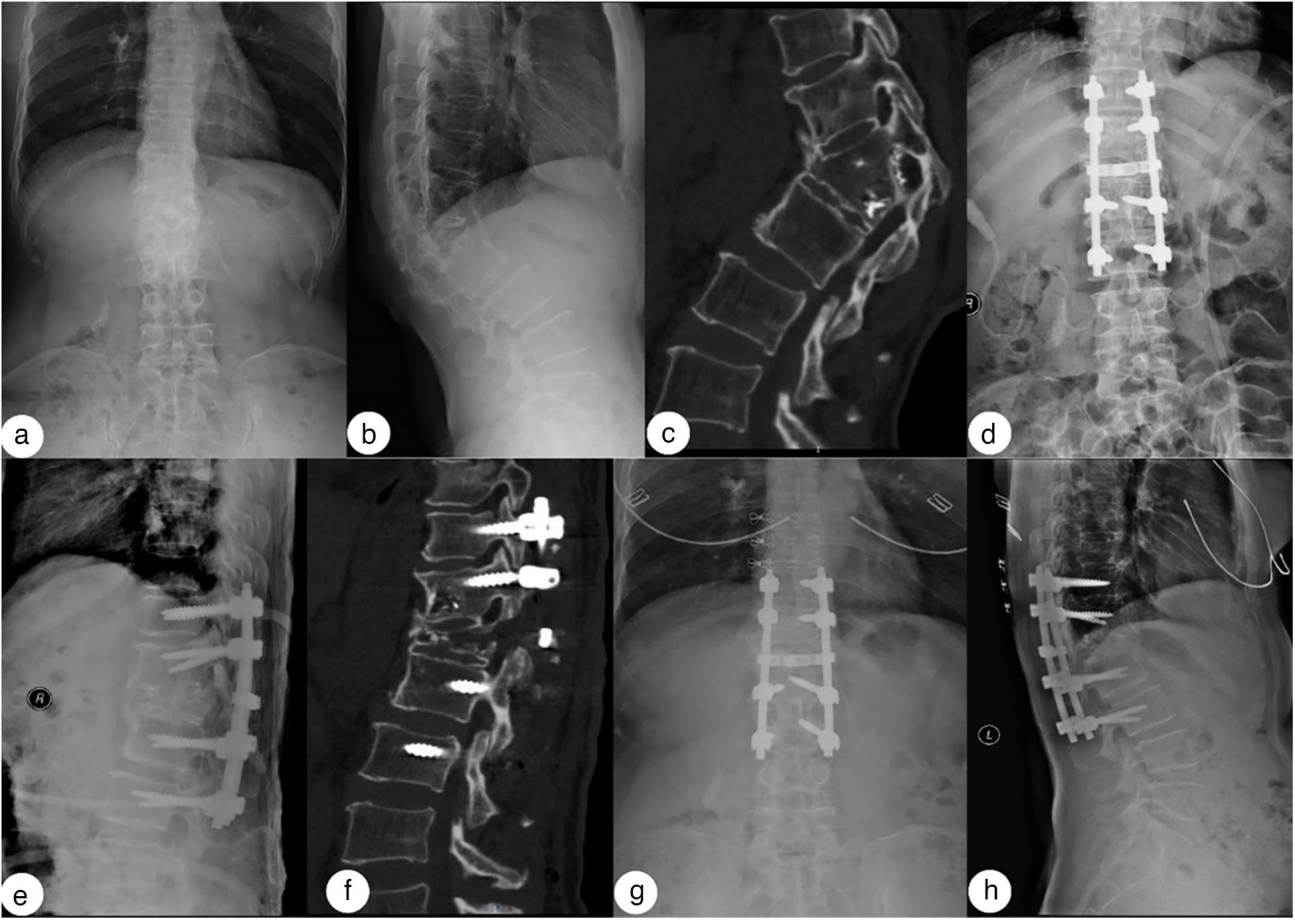
A 52-year-old woman who developed kyphosis after a T12 fracture caused by a fall. The VAS score was 9. **(a,b)** A preoperative X-ray shows a long-standing compression fracture of the T12 vertebral body, with a kyphosis Cobb angle of 54.85°. **(c,d)** Intraoperative operation and fluoroscopy. **(e,f)** Postoperative correction was satisfactory, with a kyphosis Cobb angle of 19.98°. **(g,h)** At the 1-year follow-up, there were no complications such as broken nails or rods. The osteotomy surface was fused, and the kyphosis Cobb angle was 19.67°.

**Figure 3:**
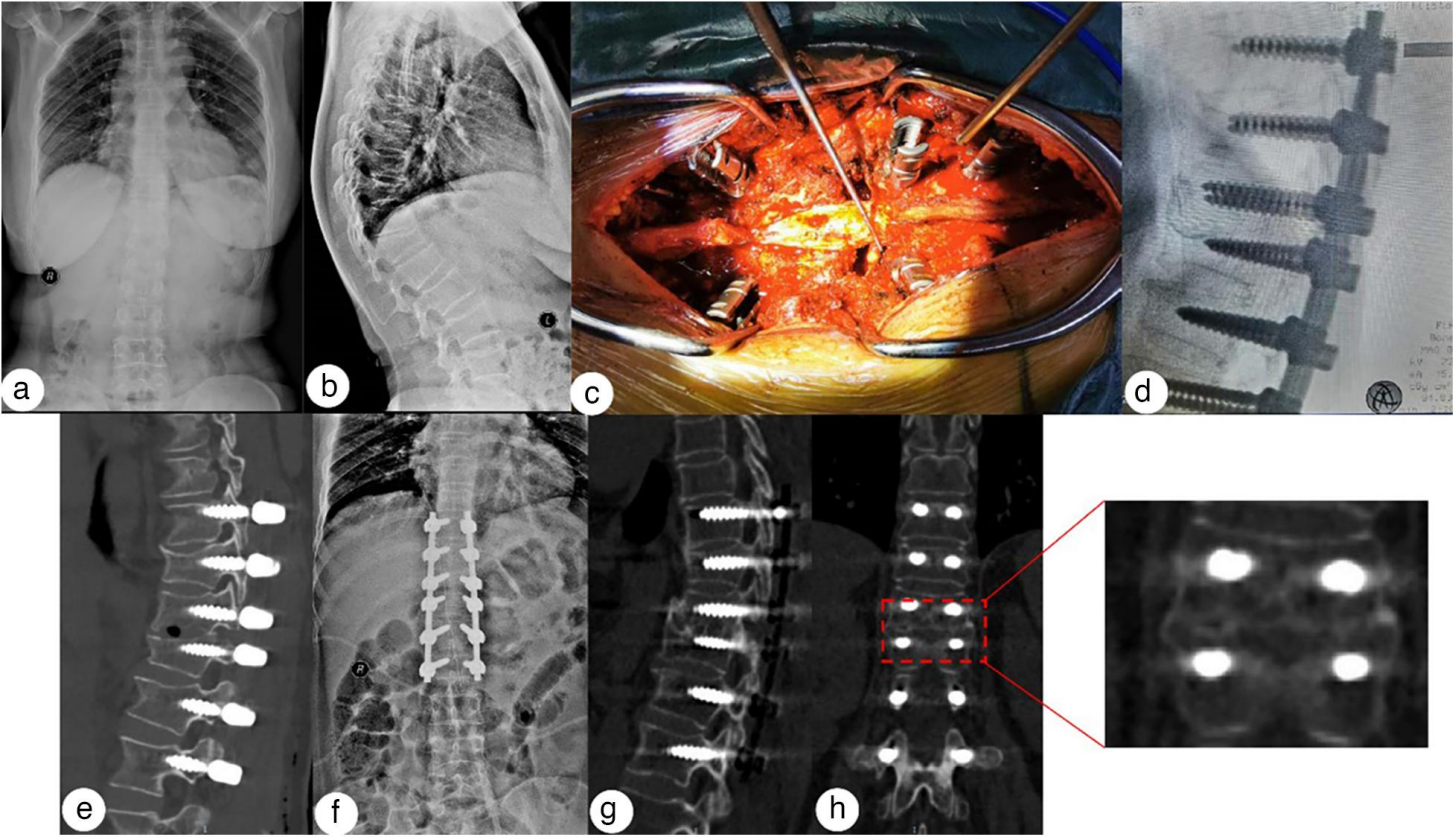
A 51-year-old woman who developed kyphosis after an L1 fracture caused by a fall. The VAS score was 8. **(a–c)** A preoperative X-ray shows a long-standing compression fracture of the L1 vertebral body, with a kyphosis Cobb angle of 61.93°. **(d–f)** The postoperative correction was satisfactory, with a kyphosis Cobb angle of 11.50°. **(g,h)** At the 2-year follow-up, there were no complications such as nail or rod breakage. The osteotomy surface was fused, and the kyphosis Cobb angle was 23.04°.

Bony fusion was observed at the last follow-up, and no internal fixation fracture, loosening, or displacement, or new vertebral fractures were noted. Typical cases are shown in Figures 2, 3.

## Discussion

Osteoporosis is defined as a BMD that is at least 2.5 SD below the average of that of healthy young women (*T* score < −2.5 SD). A systematic review and meta-analysis revealed a global prevalence of osteoporosis of 21.7% (13, 14). Osteoporosis is characterized by low bone mass and degeneration of the bone microstructure, which can significantly affect the progression of thoracolumbar vertebral deformity after fracture and bone fusion following surgery (15). In most cases, thoracolumbar vertebral fractures due to osteoporosis are caused by common daily movements (such as coughing and lifting light objects) or mild trauma (such as vehicle bumps and external impacts). These injuries are usually not treated promptly or are improperly treated (such as via massage, acupuncture, and premature weight-bearing activities), resulting in continued collapse of the fractured vertebral bodies and subsequent spinal kyphosis (16, 17).

Because adult spine curves are relatively stiff, spinal deformity in these patients typically manifests as thoracolumbar pain and neurological symptoms (18). Advances in surgical techniques have improved treatment outcomes. Schwab et al. (11) classified posterior spinal osteotomy into six levels based on previous research, among which SPO and PSO were more widely used. SPO was initially used primarily for ankylosing spondylitis, but its indications gradually expanded to include flat back syndrome and idiopathic scoliosis, and it was also used as a supplementary method for other osteotomies. Its advantages include less surgical trauma and lower risk, but the correction angle is small, and it is unsuitable for patients with moderate-to-severe sagittal plane deformities (>12 cm) (19, 20). PSO involves wedge resection of the vertebral arch and closure of the osteotomy surface using the anterior cortical bone as a hinge, which generally achieves a correction of approximately 30°–35°. It is primarily used for mild to moderate thoracolumbar kyphosis/lateral curvature deformities. Its advantages include not extending the anterior column, thereby avoiding nerve and vascular traction injury. However, the bone incision surface is larger, and the surgery time is prolonged, resulting in increased bleeding. In addition, there is a risk of torsional injury to the spinal cord due to excessive shortening of the posterior column (21).

Spinal kyphosis deformities often involve both coronal and sagittal plane abnormalities, and the corrected spine is subjected to significant deformation stress. In addition, elderly patients frequently have osteoporosis because of a lack of red bone marrow and bone metabolism disorders (22). Therefore, revision after surgical failure is not uncommon and is most frequent in cases of pseudojoint formation (i.e., bone fusion failure) (23). The use of recombinant human bone morphogenetic protein-2 has been shown to reduce the incidence of pseudojoints without increasing the risk of other complications (24). Progression of the kyphosis (curve) is another important cause of postoperative revision. Risk factors include injury to the posterior ligament complex, excessive orthosis, and long segmental fixation and fusion. The addition of transverse process hooks or vertebroplasty can reduce the incidence of proximal junctional kyphosis (25, 26). However, not all failures require surgical intervention, and the need for revision surgery should be determined based on symptoms and later progression.

In this study, it was found that there was no significant difference in the orthopedic outcomes between the PSO and the transpedicular lower vertebral space osteotomy groups. This may be related to the lack of extensive bone grafting or an interbody fusion apparatus in the latter, which can be used to maintain vertebral height and spinal cord tension in cases of angular kyphosis and other conditions. Both methods achieved satisfactory radiographic correction and clinical improvement; however, transpedicular lower vertebral space osteotomy was associated with a significantly shorter operative time and less blood loss, while the overall complication rate was numerically lower but did not reach statistical significance in this small cohort (Fisher's exact test, *P* = 0.090).

Previous studies have consistently shown that PSO, although effective for correcting rigid sagittal deformity, is associated with substantial perioperative morbidity. In a dedicated analysis of perioperative complications related to PSO, Daubs et al. reported a notable complication burden, highlighting the importance of minimizing operative time and blood loss when operating on older or medically fragile patients (27). Moreover, broader reviews focusing on complex adult spinal deformity procedures have also indicated that three-column osteotomies are accompanied by higher complication rates and greater blood loss than less invasive corrective strategies (28). In the specific setting of kyphosis following osteoporotic vertebral compression fractures, PSO-based strategies have been reported to require relatively long operative times and considerable blood loss in some series, reinforcing the rationale for exploring lower-trauma osteotomy options in osteoporotic populations (29, 30).

The extent of transpedicular lower vertebral space osteotomy includes the intervertebral discs and adjacent vertebrae and can be classified as type IV (20). This new osteotomy technique has the following advantages: (a) Preservation of the pedicle of the injured vertebra, which helps disperse stress and reduces the possibility of loosening or fracture of the internal fixation (31). (b) Destruction of the endplate can lead to a degeneration of adjacent intervertebral discs; disc removal can prevent progression of kyphosis to some extent (32, 33). (c) Ability to maintain the height of the intervertebral space following completion of the osteotomy via insertion of an interbody fusion device or bone grafting to avoid spinal cord distortion and injury. (d) Greater ease and speed, and the induction of less trauma compared with PSO. In addition, this procedure can be applied to degenerative kyphosis or infective kyphosis. For severe rigid angular kyphosis, the vertebral column resection can provide a powerful correction but has been associated with a higher overall complication rate than PSO in pooled analyses (34).

Although the new osteotomy proposed in this study aims to optimize the surgical method for correcting thoracolumbar kyphotic deformity and preliminarily confirms its effectiveness and safety, we are also clearly aware of its limitations. First, at the biomechanical level, similar to PSO, correcting the sagittal imbalance may induce or aggravate the coronal imbalance. Furthermore, in the thoracolumbar segment, the Adamkiewicz artery, originating from the root artery, is the primary nutrient vessel for the anterior spinal artery. During removal of the inferior vertebral wall, there is a risk of injury to this vessel, which may lead to catastrophic ischemic spinal cord injury. In this study, the average correction angle of transpedicular lower vertebral space osteotomy was 32.7° ± 7.3° and can be used in patients with non-ankylosing spondylitis and kyphosis Cobb angles less than 60°. We can also consider using it as a complementary operation alongside other osteotomy types, which would require further research and demonstration.

This study has several limitations. First, the retrospective, non-randomized, single-center design introduces potential selection bias, and unmeasured confounders (e.g., comorbidities, baseline frailty, and surgeon/patient preferences) may have influenced both procedure selection and outcomes. Second, the sample size was small (11 vs. 16), which may have limited statistical power and increased the risk of type II error; several baseline variables approached statistical significance (e.g., preoperative Cobb angle and ODI), and non-significant findings should therefore be interpreted cautiously. Third, follow-up intervals were not standardized, and “final follow-up” corresponded to the last available visit, which may limit the ability to draw firm conclusions about long-term mechanical complications (e.g., instrumentation loosening or recurrent kyphosis). Fourth, although we applied appropriate distribution-aware statistics and correction for multiple comparisons, repeated-measure assessment was limited by the availability of only a small number of time points. Finally, the learning curve of transpedicular lower vertebral space osteotomy was not formally evaluated; outcomes may vary with surgeon experience, and further multicenter studies are required to validate generalizability. In addition, detailed perioperative technical parameters (e.g., cement volume per screw) and comprehensive antiosteoporosis medication regimens were not uniformly recorded in the medical charts, which limited more granular analyses of their potential impact on outcomes.

## Conclusion

Our results show that, compared with PSO, transpedicular lower vertebral space osteotomy is easier and faster to perform, with shorter operative time and lower blood loss, and yields comparable radiographic correction and clinical outcomes at the last available follow-up. At this time, however, the results cannot be fully confirmed, and definitive recommendations for the use of transpedicular lower vertebral space osteotomies cannot be made until additional studies with larger patient populations are conducted.

## Data Availability

The original contributions presented in the study are included in the article/Supplementary Material, and further inquiries can be directed to the corresponding author.

## References

[1] Schousboe JT. Epidemiology of vertebral fractures. J Clin Densitom. (2016) 19:8–22. 10.1016/j.jocd.2015.08.004 26349789

[2] Delmas PD van de Langerijt L Watts NB Eastell R Genant H Grauer A Underdiagnosis of vertebral fractures is a worldwide problem: the IMPACT study. J Bone Miner Res. (2005) 20(4):557–63. 10.1359/JBMR.041214 15765173

[3] Kutsal FY Ergin Ergani GO. Vertebral compression fractures: still an unpredictable aspect of osteoporosis. Turk J Med Sci. (2021) 51:393–9. 10.3906/sag-2005-315 32967415 PMC8203169

[4] Koelé MC Lems WF Willems HC. The clinical relevance of hyperkyphosis: a narrative review. Front Endocrinol (Lausanne). (2020) 11:5. 10.3389/fendo.2020.00005 32038498 PMC6993454

[5] Kado DM Huang MH Barrett-Connor E Greendale GA. Hyperkyphotic posture and poor physical functional ability in older community-dwelling men and women: the Rancho Bernardo study. J Gerontol A Biol Sci Med Sci. (2005) 60(5):633–7. 10.1093/gerona/60.5.633 15972617 PMC1360196

[6] Lorbergs AL O’Connor GT Zhou Y Travison TG Kiel DP Cupples LA Severity of kyphosis and decline in lung function: the Framingham study. J Gerontol A Biol Sci Med Sci. (2017) 72(5):689–94. 10.1093/gerona/glw124 27341855 PMC5964740

[7] Jang H-D Kim E-H Lee JC Choi S-W Kim HS Cha J-S Management of osteoporotic vertebral fracture: review update 2022. Asian Spine J. (2022) 16(6):934–46. 10.31616/asj.2022.0441 36573301 PMC9827207

[8] Alpantaki K Dohm M Korovessis P Hadjipavlou AG. Surgical options for osteoporotic vertebral compression fractures complicated with spinal deformity and neurologic deficit. Injury. (2018) 49:261–71. 10.1016/j.injury.2017.11.008 29150315

[9] Smith-Petersen MN Larson CB Aufranc OE. Osteotomy of the spine for correction of flexion deformity in rheumatoid arthritis. Clin Orthop Relat Res. (1969) 66:6–9. 10.1097/00003086-196909000-00003 5357786

[10] Chang KW Cheng CW Chen HC Chang KI Chen TC. Closing-opening wedge osteotomy for the treatment of sagittal imbalance. Spine (Phila Pa 1976). (2008) 33:1470–7. 10.1097/BRS.0b013e3181753bcd 18520943

[11] Schwab F Blondel B Chay E Demakakos J Lenke L Tropiano P The comprehensive anatomical spinal osteotomy classification. Neurosurgery. (2014) 74:112–20. 10.1227/NEU.0000000000000182o 24356197

[12] Muñoz-Garach A García-Fontana B Muñoz-Torres M. Nutrients and dietary patterns related to osteoporosis. Nutrients. (2020) 12(7):1986. 10.3390/nu12071986 32635394 PMC7400143

[13] Kanis J.A. Cooper C. Rizzoli R., J.-Y. Reginster on behalf of the Scientific Advisory Board of the European Society for Clinical and Economic Aspects of Osteoporosis (ESCEO) and the Committees of Scientific Advisors and National Societies of the International Osteoporosis Foundation (IOF) European guidance for the diagnosis and management of osteoporosis in postmenopausal women. Osteoporos Int. (2019) 30:3–44. 10.1007/s00198-018-4704-5 30324412 PMC7026233

[14] Salari N Darvishi N Bartina Y Larti M Kiaei A Hemmati M Global prevalence of osteoporosis among the world older adults: a comprehensive systematic review and meta-analysis. J Orthop Surg Res. (2021) 16:669. 10.1186/s13018-021-02821-8 34774085 PMC8590304

[15] Jain N Labaran L Phillips FM Khan SN Jain A Kebaish KM Prevalence of osteoporosis treatment and its effect on postoperative complications, revision surgery and costs after multi-level spinal fusion. Glob Spine J. (2022) 12:1119–24. 10.1177/2192568220976560 PMC9210228 33334188

[16] Li N Cavagnaro MJ Xiong K Du X Shi J. The multi-modal risk analysis and medical prevention of lumbar degeneration, fatigue, and injury based on FEM/BMD for elderly Chinese women who act as stay-home grandchildren sitters. Front Public Health. (2021) 9:700148. 10.3389/fpubh.2021.700148 34888274 PMC8648567

[17] Papa JA. Conservative management of a lumbar compression fracture in an osteoporotic patient: a case report. J Can Chiropr Assoc. (2012) 56:29–39. PMID: 22457539; PMCID: PMC3280116 22457539 PMC3280116

[18] Heary RF Kumar S Bono CM. Decision making in adult deformity. Neurosurgery. (2008) 63:69–77. 10.1227/01.NEU.0000320426.59061.79 18812935

[19] La Marca F Brumblay H. Smith-Petersen osteotomy in thoracolumbar deformity surgery. Neurosurgery. (2008) 63:A163–70. 10.1227/01.NEU.0000320428.67620.4F 18812920

[20] Enercan M Ozturk C Kahraman S Sarier M Hamzaoglu A Alanay A. Osteotomies/spinal column resections in adult deformity. Eur Spine J. (2013) 22(Suppl 2):S254–64. 10.1007/s00586-012-2313-0 22576156 PMC3616463

[21] van Loon PJM van Stralen G van Loon CJM van Susante JLC. A pedicle subtraction osteotomy as an adjunctive tool in the surgical treatment of a rigid thoracolumbar hyperkyphosis: a preliminary report. Spine J. (2006) 6:195–200. 10.1016/j.spinee.2005.04.008 16517393

[22] La Maida GA Luceri F Gallozzi F Ferraro M Bernardo M. Complication rate in adult deformity surgical treatment: safety of the posterior osteotomies. Eur Spine J. (2015) 24(Suppl 7):879–86. 10.1007/s00586-015-4275-5 26443695

[23] Pichelmann MA Lenke LG Bridwell KH Good CR O’Leary PT Sides BA. Revision rates following primary adult spinal deformity surgery: six hundred forty-three consecutive patients followed-up to twenty-two years postoperative. Spine (Phila Pa 1976). (2010) 35:219–26. 10.1097/BRS.0b013e3181c91180 20038867

[24] Poorman GW Jalai CM Boniello A Worley N McClelland S III Passias PG. Bone morphogenetic protein in adult spinal deformity surgery: a meta-analysis. Eur Spine J. (2017) 26:2094–102. 10.1007/s00586-016-4841-5 28281003

[25] Thawrani DP Glos DL Coombs MT Bylski-Austrow DI Sturm PF. Transverse process hooks at upper instrumented vertebra provide more gradual motion transition than pedicle screws. Spine (Phila Pa 1976). (2014) 39:E826–32. 10.1097/BRS.0000000000000367 24732851

[26] Burke JF Scheer JK Lau D Safaee MM Lui A Jha S Failure in adult spinal deformity surgery: a comprehensive review of current rates, mechanisms, and prevention strategies. Spine (Phila Pa 1976). (2022) 47:1337–50. 10.1097/BRS.0000000000004435 36094109

[27] Daubs MD Brodke DS Annis P Lawrence BD. Perioperative complications of pedicle subtraction osteotomy in the lumbar spine. Global Spine J. (2016) 6(7):630–6. 10.1055/s-0035-1570088 27781181 PMC5077708

[28] Sciubba DM Yurter A Smith JS Kelly MP Scheer JK Goodwin CR A comprehensive review of complication rates after surgery for adult deformity: the role of three-column osteotomy. Global Spine J. (2015) 5(3):234–43. 10.1016/j.jspd.2015.04.005 27927561

[29] Kim SK Chung JY Park YJ Choi SW Seo HY. Modified pedicle subtraction osteotomy for osteoporotic compression fracture with kyphotic deformity. Orthop Surg. (2020) 12:372–80. 10.1111/os.12589 32107881 PMC7189028

[30] Kim S-K Chung J-Y Park Y-J Choi S-W Seo H-Y. Pedicle subtraction osteotomy for kyphosis following osteoporotic vertebral fracture. Case Rep Orthop. (2018) 2018:1–6.

[31] Cheng LM Wang JJ Zeng ZL Zhu R Yu Y Li C Pedicle screw fixation for traumatic fractures of the thoracic and lumbar spine. Cochrane Database Syst Rev. (2013) 2013(5):CD009073. 10.1002/14651858.CD009073 23728686 PMC11831250

[32] Su Y Ren D Liu D Li J Wang T Qi W Effects of endplate healing morphology on intervertebral disc degeneration after pedicle screw fixation for thoracolumbar fractures. Medicine (Baltimore). (2021) 100:e25636. 10.1097/MD.0000000000025636 33907120 PMC8084067

[33] Verlaan JJ Dhert WJA Oner FC. Intervertebral disc viability after burst fractures of the thoracic and lumbar spine treated with pedicle screw fixation and direct endplate restoration. Spine J. (2013) 13:217–21. 10.1016/j.spinee.2012.02.032 23369497

[34] Iyer S Nemani VM Kim HJ. A review of complications and outcomes following vertebral column resection in adults. Glob Spine J. (2016) 6(6):532–9. 10.4184/asj.2016.10.3.601 PMC4917782 27340543

